# Explaining significant differences in subjective and objective measures of cardiovascular health: evidence for the socioeconomic gradient in a population-based study

**DOI:** 10.1186/1471-2261-13-64

**Published:** 2013-08-30

**Authors:** Irene Mosca, Bláithín Ní Bhuachalla, Rose Anne Kenny

**Affiliations:** 1 The Irish Longitudinal Study on Ageing (TILDA), Trinity College, Dublin 2, Ireland; 2 Economic and Social Research Institute (ESRI), Dublin 2, Ireland; 3 Department of Medical Gerontology, Trinity College, Dublin 2, Ireland; 4 Mercer's Institute for Successful Ageing, St. James’s Hospital, Dublin 8, Ireland; 5 Trinity College Institute of Neuroscience, Trinity College Dublin, Dublin 2, Ireland

## Abstract

**Background:**

To assess prevalence rates of subjective and objective reports of two cardiovascular disorders (hypertension and hypercholesterolemia) for the same subset of respondents in a large-scale study. To determine whether and the extent to which the socioeconomic health gradient differed in the subjective and objective reports of the two cardiovascular disorders.

**Methods:**

Data from the first wave (2009/2011) of The Irish Longitudinal Study on Ageing were used (n = 4,179). This is a nationally representative study of community-dwelling adults aged 50+ residing in Ireland. Subjective measures were derived from self-reports of doctor-diagnosed hypertension and high cholesterol. Objective measure of hypertension was defined as: systolic blood pressure ≥140 mm Hg and/or diastolic blood pressure ≥90 mm Hg and/or on antihypertensive medication. Objective measure of hypercholesterolemia was defined as: total cholesterol ≥5.2 mmol/L and/or on cholesterol-lowering medication. Objective measures of low-density-lipoprotein cholesterol and high-density-lipoprotein cholesterol were also used. Two measures of socioeconomic gradient were employed: education and wealth. Binary and multinomial logistic and linear regression analyses were used. Analyses were adjusted for an extensive battery of covariates, including demographics and measures of physical/behavioural health and health care utilization.

**Results:**

*Prevalence of cardiovascular disorders:* prevalence of hypertension and hypercholesterolemia was significantly higher when the cardiovascular disorders were measured objectively as compared to self-reports (64% and 72.1% versus 37% and 41.1%, respectively). *Socioeconomic gradient in hypertension*: the odds of being objectively hypertensive were significantly lower for individuals with tertiary/higher education (OR, 0.74; 95% CI, 0.60-0.92) and in the highest tertile of the wealth distribution (OR, 0.77; 95% CI, 0.62-0.95). In contrast, the associations between socioeconomic status and self-reported hypertension were not statistically significant. *Socioeconomic gradient in hypercholesterolemia*: wealthier individuals had higher odds of self-reporting elevated cholesterol (OR, 1.28; 95% CI, 1.03-1.58). Associations between socioeconomic status and objectively measured hypercholesterolemia and low-density-lipoprotein cholesterol were not significant. Higher education and, to a lesser extent, greater wealth were associated with higher levels of high-density-lipoprotein cholesterol.

**Conclusions:**

Clear discrepancies in prevalence rates and gradients by socioeconomic status were found between subjective and objective reports of both disorders. This emphasizes the importance of objective measures when collecting population data.

## Background

Previous research has shown that gradients in poor health by socioeconomic status (SES) exist [1-11]. Most studies have used self-reported measures of health status and, to a lesser extent, self-reported chronic health conditions. These measures are attractive because they are relatively inexpensive to collect in large-scale surveys and a powerful independent predictor of future morbidity and mortality [12-15] and health care utilization [16,17]. Nevertheless, there are growing concerns that findings based on self-reported measures of health may be biased if such measures suffer from reporting error and, in particular, if the reporting error varies with SES. Evidence that reporting errors undermine the robustness of the findings on socioeconomic gradients (SEGs) in health has been found in the US and in Europe [18-21]. An attractive alternative is to use objective measures of health status. These measures are, however, expensive to collect and hence rarely collated in large population studies.

The contribution of this article to the literature is twofold. First, this article assesses prevalence rates of subjective and objective reports of two cardiovascular disorders (hypertension and hypercholesterolemia) in a nationally representative study of older individuals residing in Ireland (The Irish Longitudinal Study on Ageing (TILDA)). TILDA is rare in containing both subjective and objective measures of the same condition for the same respondents and in the depth and quality of the objective measures collected. Second, this article investigates whether and the extent to which the socioeconomic health gradient differs in the subjective and objective reports of the two cardiovascular disorders.

## Methods

### Source data

Data from the first wave (2009/2011) of The Irish Longitudinal Study on Ageing (TILDA) were used. This is a nationally representative study of community-dwelling adults aged 50+ (and their spouses or partners of any age) residing in Ireland. The study is closely harmonised with leading international research, including The English Longitudinal Study of Ageing (ELSA), the Survey of Health, Ageing and Retirement in Europe (SHARE) which is pan-European, and the Health and Retirement Survey (HRS) conducted in the United States.

A total of 8,504 participants were recruited to the study (8,175 aged 50+ and 329 younger partners of eligible individuals). Ethical approval was obtained from the Trinity College Dublin Research Ethics Committee, and all participants provided written informed consent. Participants first completed a computer-assisted personal interview (CAPI) in their own homes. If individuals had known or suspected dementia, they were ineligible for participation in the study. The overall response rate was 62%.

Participants attended a health centre for a comprehensive health assessment. Participants who were unable/unwilling to attend were offered a modified and partial assessment in their own home. All assessments were carried out by qualified and trained research nurses. Among other clinical parameters, cardiovascular measures were assessed and collated. Of the 8,175 participants aged 50+, 5,897 underwent an assessment (85.4% in the health assessment and 15.6% in their own home). Details of the health assessment have been reported elsewhere [22].

### Outcome variables: subjective and objective measures of cardiovascular disorders

TILDA collected data on individual self-reports of specific conditions with the general question: “Has the doctor ever told you have any of the following conditions on this card?” The diagnoses analyzed in this paper are: hypertension and high cholesterol.

TILDA also collected data on objective evidence of these cardiovascular disorders. Focusing first on hypertension, objective measurements of blood pressure were taken by TILDA research nurses in the health or home assessment. Blood pressure was measured using the OMRON™ digital automatic blood pressure monitor with arm cuff (Model M10-IT). Participants had been seated for at least 30 minutes when the measurement was obtained. Three separate readings were taken one minute apart; the first two with the respondent seated and the third immediately after the respondent stood up. The mean value for seated blood pressure from the first and second readings was used in this analysis.

Objective hypertension was defined as systolic blood pressure (SBP) ≥ 140 mm Hg and/or diastolic blood pressure (DBP) ≥ 90 mm Hg and/or on antihypertensive medication [11,23,24]. Data on medication was collected in the CAPI. Respondents were asked to show the packaging (bottle, tube, blister pack) of the medications they were taking on a regular basis to the interviewer, who then recorded the names of the medications into a computer-based medication inventory. All medications were then classified according to the WHO Anatomical Therapeutic Chemical (ATC) classification system. This ensured that drugs were classified in a standardized way, according to primary indication. We considered antihypertensive medication to include respondents taking antiadrenergic agents, diuretics, beta blocking agents, calcium channel blockers and ACE inhibitors.

Non-fasting blood samples were collected from TILDA respondents in the health or home assessment. Measurements of total cholesterol, HDL-C (high-density-lipoprotein cholesterol) and LDL-C (low-density-lipoprotein cholesterol) were taken. Respondents were defined as having objective hypercholesterolemia if: total cholesterol ≥5.2 mmol/L and/or on cholesterol-lowering medication. We considered cholesterol-lowering medication to encompasse HMG CoA reductase inhibitors, fibrates, bile acid sequestrants, nicotinic acid derivatives and other lipid modifying agents such as ezetimibe, as well as lipid lowering drugs in combination with other agents. Measurements of HDL-C and LDL-C were also used in our analyses.

### Measures of SES

Two key SES measures were used in this study: educational attainment and wealth, each divided into three groups. Education was separated into: ‘no/primary’, ‘secondary’ (junior certificate, leaving certificate or equivalent), and ‘tertiary/higher’ (diploma, first degree or higher).

Data on wealth was collected through a battery of questions on self-valuation of: current residence, properties other than current residence, cars, savings, other financial assets and other assets including business and land. Unfolding brackets were used when respondents refused or said that they “did not know” the value of their house, other financial assets or other assets. The unfolding brackets technique is often used in empirical studies to reduce item non-response on financial measures [25].

Unfortunately, unfolding brackets were not employed after the question on savings. Hence, savings were imputed for those respondents who, although did not provide an estimate of savings, provided information on all the other assets (N = 742). Multiple imputation was performed using the multiple imputation suite (mi) of commands available is STATA 12 [26]. The mi set of commands was used to generate a regression model to impute missing data based on a range of conditional covariates. The predictive mean matching method was used. This is a partially parametric model that matches the missing value to the observed value with the closest predicted mean [26-28]. A total of five nearest neighbours were included in the set of possible donors [28]. This process was repeated 20 times, creating 20 separated imputed datasets. These 20 datasets were finally combined into one dataset. The wealth gradient was then constructed by dividing respondents in tertiles.

### Other covariates

A wide battery of controls was added to the model. These included: i) demographic and socio-economic characteristics: sex, single year of age, marital status (married/cohabiting or not), current area of residence (Dublin, another town/city, rural area), whether respondent grew up in a poor family or not (self-reported); ii) risk factors associated with cardiovascular disease: smoking (current smoker, past smoker, never smoked), drinking (standard alcoholic drinks per week and CAGE (cut-annoyed-guilty-eye opener) questionnaire score) [29,30], exercise (kilocalories burnt per week doing physical activity) and waist circumference in centimetres; iii) self-report of doctor-diagnosed diabetes; iv) multiple other doctor-diagnosed cardiovascular diseases specifically angina, heart attack, congestive heart failure, stroke, ministroke or transient ischemic attack, abnormal heart rhythm, heart murmur and any other heart trouble; v) health care utilization: number of times visited a physician or a hospital in the year prior to the interview (self-reported); and vi) health insurance coverage: private (yes/no), full public i.e. free health care (yes/no), partial public (yes/no). The regressions on hypercholesterolemia also included a dichotomous variable for whether the respondent reported to have had at least one blood test for cholesterol.

### Statistical methods

Binary and multinomial logistic and linear regression analyses were used. Analyses were first adjusted only for age and sex. Subsequently, the full battery of covariates was entered simultaneously. All models used appropriate sample weights and included 4,179 observations. Analyses were performed using STATA 12 [31]. A prior level of significance was set at p ≤ 0.05 for all analyses.

First, the associations between SES and self-reported and objectively measured hypertension and hypercholesterolemia were investigated. Binary logistic regressions were used and odd ratios (ORs) and 95% confidence intervals (CIs) were reported. Then, the associations between SES and objectively measured hypertension, LDL-C and HDL-C were investigated more closely. Multinomial logistic regressions were used for objective hypertension and, for ease of interpretation, parameter estimates were converted to estimates of average marginal effects (AMEs). Linear regressions were used for objective measures of LDL-C and HDL-C and coefficients (and 95% CIs) were reported.

## Results

Baseline characteristics of the source population are shown in Table 1. The prevalence of hypertension and hypercholesterolemia was significantly higher when the cardiovascular disorders were measured objectively, for all the education and wealth categories. Focusing on the SES, the prevalence rates of self-reported and objectively measured hypertension were higher among those at the bottom of either the education or wealth ladder compared with those at the top of each classification. In contrast, the prevalence of self-reported high cholesterol and objectively measured hypercholesterolemia was higher among those with tertiary/higher education or in the third tertile of the wealth distribution.

**Table 1:** Baseline characteristics by educational attainment and wealth tertile

	**Education**			**Wealth**			**Total**
	**No/primary**	**Secondary**	**Tertiary/ higher**	**1st tertile**	**2nd tertile**	**3rd tertile**	
Unweighted sample size	1,089	1,741	1,349	940	1,172	1,325	4,179
*Outcome variables*							
Self-reported CVDs:							
Hypertension	44.8	32.8	30.8	38.4	38.2	31.5	37.0
High cholesterol	41.5	39.8	43.7	38.7	39.7	45.5	41.1
Objectively measured CVDs:							
Hypertension^a^	74.3	59.9	52.4	69.2	63.3	57.7	64.0
Hypercholesterolemia^b^	71.9	71.2	74.6	70.9	71.9	72.4	72.1
LDL-cholesterol, mean	2.8	2.9	3.0	2.9	2.9	2.9	2.9
HDL-cholesterol, mean	1.5	1.5	1.6	1.5	1.5	1.6	1.5
*Covariates*							
Male	48.8	47.9	52.6	46.8	48.3	54.4	49.1
Age, mean	68.4	60.9	60.4	64.9	62.9	61.6	63.7
Married/cohabiting	60.3	74.9	74.9	52.6	70.6	83.2	69.4
Place of residence:							
Dublin	25.1	23.7	36.2	21.5	20.4	40.0	26.5
Another town/city	25.4	28.1	25.0	30.5	28.2	19.0	26.5
Rural area	49.5	48.2	38.9	48.1	51.4	41.0	46.9
Grew up in poor family	36.0	19.2	13.9	32.4	24.9	18.1	24.6
Smoking:							
Current smoker	19.5	17.0	11.3	27.4	14.9	11.0	16.9
Past smoker	40.9	36.9	40.1	36.9	42.4	37.2	39.1
Never smoked	39.5	46.1	48.6	35.7	42.7	51.8	44.0
Drinking:^c^							
Alcoholic drinks per week, mean	4.6	6.2	6.8	5.9	5.1	6.9	5.8
CAGE score ≤1	76.7	78.0	77.1	74.7	78.0	78.8	77.4
CAGE score ≥2	8.9	12.7	15.0	11.9	11.0	13.4	11.7
Kilocalories burnt per week, mean	3596.9	4183.5	4284.5	3339.8	3778.6	4594.4	3979.1
Waist circumference, mean	97.8	95.3	94.5	97.0	96.6	95.0	96.1
Self-reported diabetes	9.5	6.3	5.2	8.5	7.0	6.2	7.3
Self-reported other CVDs, mean	0.3	0.2	0.2	0.3	0.2	0.2	0.2
Visits to physician/H last year, mean	7.2	5.3	4.7	7.3	6.2	4.6	5.9
Health insurance coverage:							
Private	33.8	63.9	81.8	26.9	52.6	84.3	55.8
Full public	72.5	39.2	22.3	71.2	50.9	22.6	48.7
Partial public	1.9	2.0	1.3	2.7	2.6	0.6	1.8
Tested for cholesterol	90.5	88.1	90.1	86.2	89.7	91.0	89.3

Values are expressed as percentages unless otherwise indicated. Sample weights are applied. Baseline characteristics for ‘Education’ and ‘Total’ are reported for N = 4,179. Baseline characteristics for ‘Wealth’ are reported for N = 3,437.

Abbreviations: *CVD* Cardiovascular disorder, *LDL* Low-density lipoprotein, *HDL* High-density lipoprotein, *H* Hospital.

^a^Defined as: systolic blood pressure ≥ 140 mm Hg and/or diastolic blood pressure ≥ 90 mm Hg and/or on antihypertensive medication (ATC codes: C02 (A-D,K,L,N), C03, C04, C07-C09).

^b^Defined as: total cholesterol ≥5.2 mmol/L and/or on cholesterol lowering medication (ATC code: C10A (A-D, X) and C10B).

^c^Questions on alcohol intake and the CAGE questionnaire were asked in the self-completion questionnaire. Hence, information on alcohol intake and CAGE score is missing for respondents who underwent the health or home assessment but did not return the self-completion questionnaire.

### SES in self-reported and objectively measured hypertension and hypercholesterolemia

Table 2 shows the associations between SES and self-reported and objectively measured hypertension (Panel 1) and hypercholesterolemia (Panel 2). Panel 1 shows that, in the analyses adjusted for age and sex, the odds of reporting doctor-diagnosed hypertension were lower for individuals with tertiary/higher education (p < 0.05) and in the highest tertile of the wealth distribution (p < 0.05). However, adjustment for the full battery of covariates reduced these associations, such that they were no longer significant. In contrast, the odds of being objectively hypertensive remained significantly lower for individuals with tertiary/higher education (p < 0.01) and in the highest tertile of the wealth distribution (p < 0.05) even after full adjustment.

**Table 2:** Associations [95% confidence intervals] between SES (education and wealth) and self-reported and objectively measured hypertension and hypercholesterolemia

	**Education**			**Wealth**		
	**No/primary**	**Secondary**	**Tertiary/higher**	**1st tertile**	**2nd tertile**	**3rd tertile**
*Panel 1*: Hypertension						
Self-reported^a^						
Adjusted for age, sex	1 [Ref]	0.83 [0.70–0.99]*	0.79 [0.65–0.95]*	1 [Ref]	1.04 [0.86–1.25]	0.84 [0.70–1.00]*
Adjusted for age, sex & covariates	1 [Ref]	0.94 [0.77–1.14]	0.92 [0.74–1.15]	1 [Ref]	1.17 [0.95–1.45]	0.99 [0.79–1.25]
Objectively measured^a^						
Adjusted for age, sex	1 [Ref]	0.83 [0.69–1.00]*	0.60 [0.50–0.73]**	1 [Ref]	0.78 [0.65–0.93]**	0.65 [0.55–0.77]**
Adjusted for age, sex & covariates	1 [Ref]	0.96 [0.79–1.18]	0.74 [0.60–0.92]**	1 [Ref]	0.86 [0.71–1.05]	0.77 [0.62–0.95]*
*Panel 2*: Hypercholesterolemia						
Self-reported^a^						
Adjusted for age, sex	1 [Ref]	0.99 [0.84–1.17]	1.18 [0.99–1.41]	1 [Ref]	0.98 [0.82–1.17]	1.25 [1.06–1.48]**
Adjusted for age, sex & covariates	1 [Ref]	0.98 [0.81–1.18]	1.13 [0.92–1.38]	1 [Ref]	0.96 [0.79–1.17]	1.28 [1.03–1.58]*
Objectively measured^a^						
Adjusted for age, sex	1 [Ref]	0.97[0.81–1.16]	1.19 [0.98–1.45]	1 [Ref]	1.10 [0.92–1.32]	1.10 [0.92–1.31]
Adjusted for age, sex & covariates	1 [Ref]	0.96 [0.80–1.16]	1.13 [0.90–1.42]	1 [Ref]	1.10 [0.91–1.34]	1.05 [0.85–1.30]

Abbreviation: *Ref* Reference category.

^a^Binary logistic regression was performed. Association is odds ratio. The full list of covariates is presented in Table 1.

*Indicates statistical significance at the 5% level.

**Indicates statistical significance at the 1% level.

Panel 2, Table 2 shows that, in the analyses adjusted for age and sex, individuals in the third tertile of the wealth distribution had higher odds of reporting doctor-diagnosed hypercholesterolemia (p < 0.01). The association remained significant even after full adjustment (p < 0.05). The association between SES and objectively measured hypercholesterolemia was not significant at 5% level.

### A closer look at SES in objectively measured hypertension, LDL-C and HDL-C

Panel 1, Table 3 shows the associations between SES and objectively measured hypertension in the multinomial logistic regression framework. Four mutually exclusive and exhaustive non-ordered outcome categories were identified: 1) not hypertensive (SBP < 140 mm Hg and DBP < 90 mm Hg and not on antihypertensive medication); 2) hypertensive and treated optimally (SBP < 140 mm Hg and DBP < 90 mm Hg and on antihypertensive medication); 3) hypertensive and treated sub-optimally (SBP ≥ 140 mm Hg and/or DBP ≥ 90 mm Hg and on antihypertensive medication); 4) hypertensive and untreated (SBP ≥ 140 mm Hg and/or DBP ≥ 90 mm Hg and not on antihypertensive medication). The full battery of covariates was employed. Panel 1, Table 3 shows that the probability of being ‘not hypertensive’ (1) was 5.5 percentage points higher for individuals with tertiary/higher education (p < 0.01) and 4.7 percentage points higher in the highest tertile of the wealth distribution (p < 0.05). At the same time, the probability of being hypertensive and untreated (4) was lower for individuals in the second (p < 0.01) and third (p < 0.05) tertiles of the wealth distribution.

**Table 3:** Associations [95% confidence intervals] between SES (education and wealth) and objectively measured hypertension, LDL-cholesterol and HDL-cholesterol

	**Education**			**Wealth**		
	**No/primary**	**Secondary**	**Tertiary/higher**	**1st tertile**	**2nd tertile**	**3rd tertile**
*Panel 1*: Objectively Measured Hypertension^a^						
1) Not hypertensive	1 [Ref]	0.7 [−3.0 to 4.5]	5.5 [1.4 to 9.7]**	1 [Ref]	2.7 [−0.9 to 6.4]	4.7 [0.7 to 8.8]*
Hypertensive, divided in 3 sub-groups:						
2) Optimally treated	1 [Ref]	−1.9 [−5.0 to 1.2]	−2.3 [−5.8 to 1.1]	1 [Ref]	1.4 [−1.8 to 4.5]	−1.6 [−5.2 to 2.0]
3) Sub-optimally treated	1 [Ref]	−0.6 [−3.7 to 2.4]	−1.1 [−4.6 to 2.5]	1 [Ref]	1.5 [−1.6 to 4.7]	1.1 [−2.6 to 4.8]
4) Untreated	1 [Ref]	1.8 [−1.6 to 5.3]	−2.2 [−6.1 to 1.8]	1 [Ref]	−5.6 [−9.3 to −1.9]**	−4.3 [−8.3 to −0.3]*
*Panel 2*: Objectively Measured Cholesterol^b^						
LDL-C						
Adjusted for age, sex	1 [Ref]	−0.02 [−0.09-0.05]	0.04 [−0.04-0.12]	1 [Ref]	0.01 [−0.07-0.10]	0.02 [−0.06-0.10]
Adjusted for age, sex & covariates	1 [Ref]	−0.03[−0.10-0.05]	0.00[−0.08-0.09]	1 [Ref]	0.02 [−0.07-0.10]	0.00 [−0.09-0.09]
HDL-C						
Adjusted for age, sex	1 [Ref]	0.05 [0.02-0.08]**	0.11 [0.07-0.15]**	1 [Ref]	0.02 [−0.01-0.06]	0.09 [0.06-0.12]**
Adjusted for age, sex & covariates	1 [Ref]	0.02 [−0.01-0.05]	0.05 [0.01-0.09]**	1 [Ref]	0.00 [−0.03-0.03]	0.03 [0.00-0.07]

Abbreviation: Ref, reference category.

^a^1): SBP < 140 mm Hg and DBP < 90 mm Hg and not on antihypertensive medication (36%); 2): SBP < 140 mm Hg and DBP < 90 mm Hg and on antihypertensive medication (25.5%); 3): SBP ≥ 140 mm Hg and/or DBP ≥ 90 mm Hg and on antihypertensive medication (19.1%); 4): SBP ≥ 140 mm Hg and/or DBP ≥ 90 mm Hg and not on antihypertensive medication (19.3%). Multinomial logistic regression was performed, adjusting for age, sex, and covariates. Association is average marginal effect.

^b^Linear regression was performed. Association is coefficient.

*Indicates statistical significance at the 5% level.

**Indicates statistical significance at the 1% level.

Panel 2, Table 3 displays the associations of SES with objectively measured LDL-C and HDL-C in the linear regression framework. The associations of education (and wealth) and LDL-C were not significant at 5% level. However, higher education and, to a lesser extent, greater wealth were associated with higher levels of HDL-C. In the analyses adjusting for age and sex, the coefficient of ‘tertiary/higher education’ was 0.11 (p < 0.01) and decreased to 0.05 (p < 0.01) after full adjustment.

## Discussion

Cardiovascular disease is the leading cause of death both in Ireland and Europe [32]. Our analysis revealed that 64% of the older Irish population were objectively hypertensive. A prevalence of 64% is somewhat high compared to international prevalence rates when one considers our sample is relatively young (59.7% are in the 50–64 age-group). A review published in 2005 investigated the global burden of objective hypertension through analysis of worldwide data and found that in established market economies the prevalence rate of hypertension for the 50–59 and the 60–69 age groups was 44.8% and 60.3% respectively for men and 42% and 58.7% for women [24]. Crucially this study defined objective hypertension as we did (SBP ≥ 140 mm Hg and/or DBP ≥ 90 mm Hg and/or on antihypertensive medication). This underlines the fact that while it was not feasible to define objective hypertension as per the ESC guidelines ‘at least 2 blood pressure measurements per visit and at least 2 to 3 visits’ [33], our method was validated and has been used in other international studies estimating hypertension prevalence.

Another key finding of our study was the discrepancy between self-reported and objective prevalence of hypertension (37% and 64%, respectively). One could suggest that the self-reported and the objective measures are not contemporary (i.e. in the past one truly did not have hypertension but in the interval has developed it) and this induces discrepancy. However on analysis 90% of the sample had attended their physician or hospital in the past year.

A study based on data from the Health Survey for England found that objective hypertension (similarly defined as ≥140/90 or being on treatment for blood pressure) was observed in 30% of individuals of which 34% were unaware of the diagnosis [23]. The ‘lack of awareness’ figure is comparable to our findings however we find much higher objective prevalence, the corollary of which is that the impact of such ‘unawareness’ is much more sinister in terms of public health. Whether the crux of the problem is that hypertension is not simply being diagnosed/is suboptimally managed or whether poor patient awareness is the main factor, is not clear. Is there a socioeconomic reason for the discrepancy? Given that previous studies have suggested that hypertension control rates in Europe are low [23], we attempted to gain clearer understanding by disentangling the proportions of those on antihypertensive medication in the context of socioeconomic gradient.

We discovered that of our entire sample 36% were not hypertensive, 26% were appropriately managed, 19% were not treated to target and finally 19% were untreated. As illustrated in Table 3 and discussed in the results section, we found that individuals who had tertiary/higher education and in the highest tertile of the wealth distribution had a higher probability of being ‘healthy’. Interestingly optimal and suboptimal management of hypertension (the latter which could also equate to non compliance with medication) did not demonstrate a SEG although those in the middle and highest tertiles of the wealth distribution did have a lesser probability of being hypertensive and untreated. In analyses not detailed here, we tested whether the discrepancy in our results between objective hypertension and self-report of hypertension was influenced by a SEG in accuracy of self-report. As per Johnston *et al*[19] (who also demonstrated large discrepancy) those at the top of the education or wealth ladder were less likely to report false negatively (p < 0.05).

Focusing then on hypercholesterolemia, there was once again a demonstrated discrepancy between the self-report and objective measure, prevalence being 41.1% and 72.1% respectively. In terms of SEG, the wealthier were more likely to self-report elevated cholesterol however this association was not sustained objectively. Commercially there is widespread advertisement of low cholesterol spreads and it is likely this raises public awareness of hypercholesteraemia over hypertension. Interpreting self-report as patient awareness, this suggests perhaps that these individuals have been influenced by the media and are more likely to seek and pay for screening than lower socioeconomic classes.

The more educated did demonstrate a higher probability of having elevated HDL-C, which represents a more ‘positive’ lipid profile. Banks *et al.*[11] and Muennig *et al*. [34] used data from 1999/2002 NHANES in similar studies to ours, the former looking at HDL-C and the latter at both LDL-C and HDL-C. Their results also demonstrated no SEG in LDL-C. Despite no SEG, we found that a high proportion of our population have elevated LDL-C, also a primary target of dyslipidemia therapy to reduce cardiovascular risk.

It is difficult to explain the SEG in HDL-C given that we adjusted for use of cholesterol-lowering medication in separate analyses and this made no difference. One could argue that this was because participants were not fasting. However we have previously demonstrated that there was no statistically significant difference between fasting and non-fasting samples in population studies.

Limitations of this study exist in relation to our cardiovascular measures. Our methodology for diagnosis of objective hypertension, while used in other extant large scale population studies including the Health and Retirement Study (HRS) and the English Longitudinal Study on Ageing (ELSA), is not in adherence with either the American Heart Association, nor the European Cardiology Society guidelines for diagnosis of hypertension, according to which diagnosis should be based on blood pressure measurements taken repeatedly over a period of time. It must be therefore acknowledged that it is possible our prevalence levels may be an overestimation, and include those with ‘white-coat hypertension’ and also those who are on antihypertensive medications for reasons other than hypertension. Nevertheless we do not consider that this nullifies the large discrepancy we have illustrated in prevalence rates between self-report and objective hypertension. In terms of objective lipid profile, the samples were not fasting, which is the gold standard, however as discussed above we do not consider that this altered the measure significantly.

Strengths of the study are the large nationally representative sample size and the availability both self-reported and objective cardiovascular measures. This enabled more insightful investigation into the epidemiology of these prevalent health conditions.

## Conclusions

In conclusion we surmise that hypertension has a higher prevalence in Ireland than in other European countries and that Ireland fares poorly at achieving blood pressure targets in those on treatment. Lower SES groups are at more risk of hypertension and should be aggressively screened and if commenced on medication our results suggest being less wealthy or less educated will not influence likelihood of achieving treatment targets. This SEG in health is more evident using objective measures. There likely is a combination of poor patient awareness (which itself demonstrates a SEG) and undiagnosed hypertension contributing the discrepancy between self-report and objective measures. In terms of abnormal lipid profile, we interpret that there was no SEG although in analyses not detailed here we found that those in the highest tertile of the wealth gradient were more likely to report having high cholesterol when they truly did (p < 0.10).

## Competing interests

The authors declare that they have no competing interests.

## Authors’ contributions

IM, BNB and RAK were responsible for study concept and design. RAK supervised the study. IM analysed the data. IM and BNB drafted the manuscript. BNB, RAK and IM were involved in critical revision of the manuscript for important intellectual content. All authors read and approved the final manuscript.

## Pre-publication history

The pre-publication history for this paper can be accessed here:

http://www.biomedcentral.com/1471-2261/13/64/prepub
